# Synergistic effects of *Trichoderma* and biochar on the biocontrol of two soil-borne phytopathogens in chickpeas

**DOI:** 10.3389/fmicb.2025.1583114

**Published:** 2025-05-01

**Authors:** Ranjna Kumari, Vipul Kumar, Bhupendra Koul, Mohammad Abul Farah, Awdhesh Kumar Mishra

**Affiliations:** ^1^Department of Botany, Lovely Professional University, Phagwara, Punjab, India; ^2^Department of Plant Pathology, School of Agriculture, Lovely Professional University, Phagwara, Punjab, India; ^3^Department of Biotechnology, Lovely Professional University, Phagwara, Punjab, India; ^4^Department of Zoology, College of Science, King Saud University, Riyadh, Saudi Arabia; ^5^Department of Biotechnology, Yeungnam University, Gyeongsan, Republic of Korea

**Keywords:** FTIR-ATR spectroscopy, spectral signature, SEM, *Trichoderma*, biochar (BC), phytopathogens, chickpea

## Abstract

**Introduction:**

This study aims to identify and characterize four *Trichoderma* isolates using molecular techniques, Fourier transform infrared spectroscopy (FTIR), and volatile organic compounds (VOC) profiling.

**Methods:**

The antagonistic activity of these isolates was assessed against *Fusarium oxysporum* f. sp. *ciceri* (FOC) and *Sclerotium rolfsii* (SR) using a dual culture technique. The synergistic effect of *Trichoderma harzianum* (accession no. PP256488) combined with biochar (BC) was evaluated for plant growth enhancement and disease suppression. Four *Trichoderma* isolates (*T. harzianum, T. asperellum, T. virens*, and *T. lixii*) were identified through ITS region analysis, VOC profiling, and FTIR spectroscopy.

**Results:**

Molecular analysis confirmed their distinct identities, and GC-MS analysis revealed 37 VOCs out of 162 with antipathogenic properties. Unique FTIR peaks were recorded at 3271.96 cm^−1^ for *T. virens*, 2800–2900 cm^−1^ for *T. asperellum*, and 2850–2950 cm^−1^ for both *T. lixii* and *T. harzianum*. Scanning electron microscopy (SEM) analysis of *T. harzianum* revealed mycoparasitic structures, including hyphal coils, penetration holes, and appressoria, indicating effective pathogen interaction. The combined application of *Trichoderma* and biochar (T9) significantly enhanced root length (9.23 cm), plant height (26.03 cm), and root mass (43.33 g) in chickpea plants. Moreover, treatments (T9) and (T10) reduced the disease incidence in chickpeas, decreasing fusarium wilt by 27% and collar rot by 33%, respectively.

**Conclusion:**

This sustainable approach exhibits the potential of combined application of *Trichoderma* and biochar which can enhance plant growth and reduce disease incidence, and improve food security.

## 1 Introduction

Chickpea (*Cicer arietinum* L.), a member of the Fabaceae family, is the second most widely grown legume worldwide after beans and is grown in agricultural fields across more than 50 countries. Also known as “garbanzo beans,” chickpeas are rich in dietary fiber, protein, and essential vitamins, including A, C, E, K, B-complex, and omega-6 (linoleic acid), as well as minerals such as calcium (Ca), zinc (Zn), magnesium (Mg), and iron (Fe) (Mula et al., 2011; Koul et al., 2022; Jukanti et al., 2012). Additionally, chickpeas are recognized as a sustainable and climate-resilient crop, ranking among the most traded pulses worldwide (Vadez et al., 2012). Their drought tolerance makes them a crucial source of income for farmers in areas with limited water supplies (Mula et al., 2011). However, the vigor and viability of chickpea crops are negatively affected by biotic and abiotic environmental factors. Several soil-borne phytopathogens adversely influence the vegetative growth and yield of chickpeas, including *Sclerotinia sclerotiorum, Sclerotium rolfsii, Fusarium oxysporum, Macrophomina phaseolina*, and *Rhizoctonia solani* (Mageshwaran et al., 2022; Yaseen and Mukhtar, 2024). The resting structures (e.g., sclerotia and chlamydospores) formed by the soil-borne pathogens *Sclerotium rolfsii* and *Fusarium oxysporum* pose significant threats to chickpea production (Maleki et al., 2024). To control these pathogens, several routine methods are employed: (i) using resistant varieties, (ii) early sowing, (iii) alternate cropping, (iv) seed treatment with fungicides, and (v) application of biocontrol agents (BCAs) such as *Pseudomonas* spp., *Bacillus* spp., and *Trichoderma* spp. (Landa et al., 2004; Leisso et al., 2009).

Among these, *Trichoderma* is an effective biocontrol agent and plant growth-promoting fungus (PGPF) (Araujo et al., 2019; Abuhena et al., 2023; Manzar et al., 2024). It possesses several beneficial traits, including the ability to sense and penetrate host tissues, produce bioactive compounds such as indole acetic acid, 1-aminocyclopropane-1-carboxylate, and siderophores, and protect crops from pathogens (Poveda et al., 2023; Contreras-Cornejo et al., 2024). *Trichoderma* employs biocontrol mechanisms both directly and indirectly against phytopathogens. Direct mechanisms include mycoparasitism, competition for space and nutrients, and antibiosis. Indirect mechanisms involve stimulating plant growth, inducing microbial resistance, enhancing root colonization efficiency, and bioremediation (Benítez et al., 2004; Kumari et al., 2024).

*Trichoderma* spp. are often isolated from habitats such as rhizospheric soil, crop residue, water, and farmyard manure (Sharma et al., 2019). To identify a new strain, a precise and specific description of the fungi is essential (Abdenaceur et al., 2024). Identifying *Trichoderma* based on morphological characteristics poses challenges due to minimal variation in cultural and morphological traits. Thus, molecular identification, including DNA sequencing, is necessary for the accurate identification of *Trichoderma* at the species level (Sacchi et al., 2002). Routine techniques for this purpose include morphological and microscopic identification, which are time-consuming and require proficiency (Homechin and de Azevedo, 1987), as well as molecular characterization utilizing PCR and genome sequencing of the ITS (internal transcribed spacer) region or another specific gene (β-tubulin) (Kredics et al., 2018; Ezeonuegbu et al., 2022; Zandyavari et al., 2024). However, while these techniques are highly reliable and advantageous, they are also costly and require expertise.

Fourier transform infrared spectroscopy (FTIR) is a reliable method for characterizing and identifying various microbes, particularly fungi (Naumann et al., 2005; Lecellier et al., 2015). Several reports have documented variations among fungi at both the genus and species levels (Erukhimovitch et al., 2010). This spectral signature correlates with various biomolecules, including genetic materials (DNA and RNA), polysaccharides, fats, proteins, and lipids in the biomass of microorganisms (Essendoubi et al., 2007). FTIR is primarily used for measurements in the mid and near-IR (infrared) regions. According to Almoujahed et al. (2024), the MIR (medium infrared region; 650–4000 nm) and NIR (near-infrared regions; 750 to 2,500 nm) regions of the FTIR spectrum effectively identify and differentiate fungal species. This technique reduces the amount of time required to prepare samples and can be applied in agriculture to identify plant species and distinguish soil-borne diseases (Ghareeb et al., 2024). This technology is emerging in various fields of agriculture, including the identification of grass species (Kang et al., 2023), distinguishing soil-borne pathogens from the genus *Fusarium* (Rampersad, 2020), detection of *Trichoderma* metabolites (Guo et al., 2022), and identification of airborne fungal pathogens (Nageen et al., 2021).

In microbial studies, FTIR spectroscopy is particularly valuable for identifying functional groups in biological samples. For instance, absorption peaks in the range of 900–2000 cm^−1^ correspond to polysaccharides in fungal cell walls and help in distinguishing different species (Rodriguez-Saona and Allendorf, 2011). While FTIR has been widely utilized for bacterial identification, its application in characterizing biocontrol fungi, such as *Trichoderma*, is still developing (Fantin et al., 2022; Quezada et al., 2024). However, its speed, affordability, and sensitivity make it a promising tool in plant pathology (Kummerle et al., 1998; Kumar et al., 2021). It has become an attractive technique for the characterization of fungal species due to the existing information regarding the spectral signatures derived from FTIR spectra of viable cells (Diem et al., 1999). With growing research, FTIR could emerge as an essential method for distinguishing beneficial fungi in agriculture (Barrera-Patiño et al., 2023; Szymańska-Chargot et al., 2024).

Characterizing *Trichoderma* species through volatile compounds such as hydrocarbons, aromatics, amines, thiols, and terpenes involves analyzing the unique blend of volatile organic compounds (VOCs) emitted by different *Trichoderma* strains. These distinct VOC profiles enable the differentiation and identification of various *Trichoderma* species, providing insights into their potential applications in agriculture and biocontrol strategies (Lee et al., 2016). *Trichoderma*-derived secondary metabolites are particularly notable for their strong antifungal activity, especially against pathogens like *Fusarium oxysporum*. These metabolites disrupt hyphal integrity and reduce the virulence of the pathogen, contributing to their biocidal function (Zhang et al., 2024). Hasnain et al. (2024) showed the potency of *Trichoderma* metabolites in inhibiting Fusarium growth, minimizing wilt incidence in peas to 27.08% under greenhouse conditions and 28.15% in field conditions. Likewise, in cotton, the volatile compounds from *Trichoderma* spp. demonstrated 54.9% suppression of fungal wilt caused by *Fusarium oxysporum* f.sp. *vainfectum*, while the treatment also enhanced plant biomass (Mahmood et al., 2024). Similarly, Mustafa et al. (2024) examined the effect of plant extract against *Fusarium* wilt in tomatoes, highlighting this eco-friendly approach as a potential alternative for controlling plant diseases.

In our recent report, 21 *Trichoderma* isolates from various rhizospheric soils were assessed for their effectiveness against FOC and SR, which respectively cause wilt and collar rot in chickpeas (Kumari et al., 2024). Among these, isolates PBT13 and PBT3 exhibited the highest inhibition rates against the pathogens, with percentages of 72.97% and 61.1% for PBT13 and 72.23% and 59.3% for PBT3, respectively. The criteria for selecting effective *Trichoderma* isolates included mycelial extension rate, confrontation assays, and the production of hydrolytic enzymes and bioactive compounds identified through GC-MS analysis. Four isolates (PBT3, PBT4, PBT9, and PBT13) demonstrated significant antagonistic activity, produced antifungal metabolites, and exhibited high levels of chitinase and β-1,3-glucanase. Molecular characterization identified these isolates as *T. virens, T. asperellum, T. lixii*, and *T. harzianum* (Kumari et al., 2024).

*Trichoderma* has been extensively studied as a biological control agent (BCA) (Woo et al., 2014; Kumari et al., 2024). However, a single strain of *Trichoderma* is less effective in controlling soil-borne diseases under greenhouse and field conditions (Trivedi et al., 2020; Manzar et al., 2022). The use of multiple strains of *Trichoderma* has recently gained attention (Kredics et al., 2024). By stimulating the synthesis of secondary metabolites and silent genes, the combined use of several species may further support plant development and provide defense against plant diseases (Netzker et al., 2015; Knowles et al., 2022). Nonetheless, optimizing application ratios, efficacy, and inoculation techniques poses significant challenges associated with the co-application of different *Trichoderma* strains (Liu et al., 2022). Therefore, to enhance their bio-efficacy, these BCAs can be utilized alongside biochar (BC). BC serves as a potential carrier for beneficial microbial inoculants due to its various beneficial properties, such as high porosity, water-holding capacity, specific surface area, nutrient availability, and a wide variety of functional groups (hydroxyl, carboxyl, sulfonic acid groups, and so on) (Zhang et al., 2020). Numerous studies have reported increased microbial activity and improved soil health with BC as an amendment (Bolan et al., 2024). The co-application of both beneficial microorganisms and BC effectively suppresses phytopathogens while also contributing to the colonization of microbes. Graber et al. (2010) found that soil amended with BC enhanced the *Trichoderma* population compared to non-amended soils. Hu et al. (2014) also reported a significant change in the fungal community after the addition of BC to the soil, showing a *Trichoderma* population that was 14.5% larger than that in non-amended soil. Thus, this approach is emerging for crop protection. Moreover, de Medeiros et al. (2021) reported the combined use of *Trichoderma* and BC to specifically prevent soil-borne phytopathogens (*Fusarium* spp., *Sclerotinia sclerotiorum*, and *Macrophomina phaseolina*). However, research findings on the use of BC coupled with *Trichoderma* for controlling soil-borne diseases and its mechanism of action are limited (Liu et al., 2023).

The objective of this study was to characterize *Trichoderma* strains using molecular analysis, FTIR spectroscopy, and VOC profiling techniques. Among the four species, the one with the best biocontrol efficacy was selected and combined with BC as a carrier material. This combination was evaluated for its synergistic effect on the growth of chickpea (*Cicer arietinum* L.) plants infected with two significant soil-borne diseases: wilt and collar rot, caused by *Fusarium oxysporum* f. sp. *ciceri* and *Sclerotium rolfsii*, respectively. Moreover, understanding the synergistic potential of BC and *Trichoderma* for the biocontrol of these soil-borne pathogens and for promoting chickpea growth is essential.

## 2 Materials and methods

### 2.1 *Trichoderma* strains

Four *Trichoderma* strains—PBT9 (*T. lixii*), PBT13 (*T. harzianum*), PBT4 (*T. asperellum*), and PBT3 (*T. virens*)—were isolated from 21 different rhizospheric soil samples collected from uncultivated fields in Punjab, India (Kumari et al., 2024). To isolate the *Trichoderma* strains, 1g of rhizospheric soil was suspended in 90 mL of autoclaved water within 100 mL borosilicate conical flasks. These flasks were placed on a vortex mixer set at 200 rpm for 15 min to ensure thorough homogenization. Following this, the samples underwent serial dilution up to 10^−6^ (Akhtar et al., 2022). A 100 μL suspension of each sample was spread onto *Petri* dishes containing Rose Bengal agar (RBA), a selective medium, and the plates were incubated at 25°C for 3 days. A single colony was subsequently transferred to potato dextrose agar (PDA) plates to obtain a pure culture (Shrestha et al., 2024). Based on their macro- and micromorphological features—including pigmentation, conidiophore structure, and conidial shape—the isolates were classified as members of the genus *Trichoderma*. The plates were stored in a refrigerator at 4°C for future use.

### 2.2 Characterization of *Trichoderma* strains: molecular, biochemical, and VOC profiles

Four strains of *Trichoderma*, namely *T. lixii, T. harzianum, T. virens*, and *T. asperellum*, were used in this study to examine their variation. These species were maintained in mineral oil and on agar slants and stored in a refrigerator at 4°C. A monosporic culture was utilized to develop a pure culture of the isolates (Hewedy et al., 2020).

#### 2.2.1 Molecular characterization

DNA isolated from *Trichoderm*a was extracted using a DNA isolation kit (Thermo Fisher Scientific, USA). A 5 mm disc of a 7-day-old fungal culture was introduced into the PDB medium and maintained at 28°C for 72 h. The mycelial mat was harvested with Whatman filter paper, and ~200 mg of mycelium was crushed using a sterilized mortar and pestle to extract DNA (Doyle, 1987; Abraham et al., 2017). The extracted DNA was re-suspended in TE buffer (pH 8.0), and its concentration and integrity were determined by Nanodrop analysis and visualization on a 1% agarose gel, respectively. ITS1 and ITS4 primers were used for ITS region amplification following the protocol described by White et al. (1990). PCR was performed in a 100 μL reaction containing *Taq* polymerase (1 unit), MgCl_2_ (1.5mM), primers (0.2μM), dNTPs (0.2mM), and DNA (100ng). PCR amplification was conducted using a thermocycler (Eppendorf, Germany) with an initial denaturation at 94°C for 10 min, followed by 30 cycles (94°C for 30 s, 52°C for 30 s, 72°C for 30 s) and a final extension at 72°C for 5 min. PCR products were stained with ethidium bromide, subjected to 1% agarose gel electrophoresis, and detected using UV transillumination. DNA was purified using the PureLink Quick Gel Extraction Kit (Takara Bio, Japan), assessed by Nanodrop, and sent to UNESP/Jaboticabal, Brazil, for sequencing (ABI 3,500, Applied Biosystems) (Abraham et al., 2017).

#### 2.2.2 Phylogenetic analysis of *Trichoderma* species

The genetic sequences of various *Trichoderma* species were retrieved from the GenBank database, and their phylogenetic relationships were analyzed. Species identification was based on the highest identity matches, with a maximum query coverage of 100% (Al-Salihi and Alberti, 2023). MEGA XI was employed to study the evolutionary relationships, and the resulting DNA sequences were submitted to GenBank with the following accession numbers: *T. virens* (ON678281), *T. asperellum* (PP256386), *T. harzianum* (PP256488), and *T. lixii* (PP256388).

### 2.3 Biochemical characterization

#### 2.3.1 FTIR analysis

Four *Trichoderma* samples were prepared according to Fantin et al. (2022) and analyzed using molecular methods and Fourier Transform Infrared Spectroscopy with Attenuated Total Reflectance (FTIR-ATR) (Fantin et al., 2022). Cultures were grown on PDA at 25°C for 4 days in a biological oxygen demand (BOD) incubator. A 0.5 g sample (mycelium) of 4-day-old *Trichoderma* was mixed with 10 mL of 50% ethanol, 99.5% ethanol, or ultrapure water in airtight flasks and shaken at 200 rpm for 1 h. The suspension was filtered using Whatman No. 1 paper, extracted with *n*-hexane (10 mL), and centrifuged twice at 10,000 rpm for 10 min. A total of 24 samples (three replicates of four isolates) were prepared. Hemocytometer calibration ensured uniform concentrations. FTIR-ATR spectra were recorded using 500 μL of each sample, with 32 scans across the range of 650–4,000 cm^−1^ at a resolution of 4 cm^−1^ (Durak and Depciuch, 2020).

#### 2.3.2 Pre-processing of spectrum

The whole spectrum was divided into three characterization zones (I, II, and III). Each zone contained a different multivariate model that corresponded to the absorption wavelength range of the *Trichoderma* samples. The spectrum profiles were normalized using multiplicative signal correction (MSC). For data analysis, distinct spectral areas were selected within the range of 600–4000 cm^−1^. To evaluate the similarity between *Trichoderma* samples, pre-processed spectra underwent principal component analysis (PCA) and hierarchical cluster analysis (HCA). The Euclidean distance metric and the Ward distance clustering technique were employed to create HCA plots, commonly referred to as dendrograms. The clusters in the dendrogram represent the similarity of biochemical traits present in individual samples. Unscrambler X software (CAMO, Sweden) was utilized during the preprocessing and multivariate analysis phases (Yousuff and Babu, 2023).

### 2.4 Volatile organic compound (VOC) profiling

To prepare the crude *Trichoderma* extract, four isolates were inoculated into four separate 250 mL conical flasks, each containing 100 mL of PDB, and incubated in a biological oxygen demand (BOD) incubator for 9 days at a temperature of 28 ± 2°C. Whatman filter paper No. 41 was utilized to filter the cultures after incubation, removing mycelium, hyphae, and other fragments to obtain the crude extract of secondary metabolites. A control experiment was conducted by placing 30 μL of the final filtrate onto a PDA plate to ensure the absence of conidia and mycelia, thus verifying the effectiveness of the filtration process. *Petri* dishes that had been incubated at 28 ± 2°C for 3 days were checked for *Trichoderma* growth. Secondary metabolites (bioactive compounds) were extracted using the solvent extraction method, employing a 1:1 mixture of ethyl acetate (organic solvent) and culture filtrate (the microbial broth extract). The upper layer of solvent, enriched with compounds, was separated from the aqueous PDB medium using a separating funnel. The ethyl acetate in the filtrate was removed using a rotary vacuum evaporator (Aditya Scientific Technologies, India) operated at 40°C and 70 rpm, continuing the process until the concentrate was reduced to a final volume of 4 mL. The extract was then stored in a deep freezer set at −20°C. The sample extracts were analyzed using gas chromatography-mass spectrometry (GC-MS), as reported earlier (Kumari et al., 2024).

### 2.5 SEM analysis of hyphal interaction between *Trichoderma* and phytopathogens

Scanning electron microscopy (SEM) was employed to investigate the hyphal interaction between *Trichoderma* and phytopathogens [*Sclerotium rolfsii* (SR) and *Fusarium oxysporum* f.sp. *ciceri* (FOC)]. These pathogens (SR ITCC no. 8527 and FOC ITCC no. 6341) were obtained from the Indian-Type Culture Collection (ITCC) in New Delhi, India, for this research. A 5 mm mycelium disc from each fungal *Petri* dish (phytopathogens and *Trichoderma*) was inoculated opposite one another at the edge of the *Petri* dish to establish hyphal contact between the pathogen and the antagonist (*Trichoderma*). Both phytopathogens were also plated separately to serve as controls, and the plates were incubated in a BOD incubator set at 28 ± 2°C. Agar discs (~2 mm^2^) were harvested from the dual culture at the points where both fungi were in contact. A sterilized razor blade was employed to remove excess agar before sample preparation. The mycelium disc was fixed in 2.5% glutaraldehyde overnight. The sample was washed in a gradient of ethanol (100%) at ambient temperature for 5 min. Subsequently, the sample was freeze-dried and analyzed using SEM (Long et al., 2023).

### 2.6 Preparation of BC

The BC was prepared from hardwood using an electric tubular furnace. The hardwood biomass was procured from the LPU (Lovely Professional University, Phagwara) agricultural field (31.2560° N, 75.7051° E). It was oven-dried overnight (12 h at 80°C), ground, and stored in an airtight container to create an oxygen-deficient environment before transferring to an electric tubular furnace (Nabertherm, Germany), which was heated to 500°C for 4 h. The synthesized BC was sieved using various mesh sizes (100, 200, and 300 mm) (Cao et al., 2011).

#### 2.6.1 Inhibitory and synergistic effects of BC

The food poisoning technique was employed to evaluate both the direct and synergistic effects of BC on the growth of soil-borne pathogens (FOC and SR) and *Trichoderma*. The PDA growth medium was prepared with five different concentrations of BC (1, 2, 3, 4, and 5%) and poured into *Petri* dishes in triplicate. The non-inoculated media served as the control. A small piece (7 mm) of pathogens and *Trichoderma* was placed into each *Petri* dish. The samples were sealed with parafilm tape and incubated in a BOD incubator for 96 h. After 96 h of incubation, the mycelium growth rate (in mm) was measured (Araújo et al., 2018). The serial dilution technique was applied to assess the synergistic effect of BC on the *Trichoderma* population under field conditions (Paveen et al., 2024).

### 2.7 Pathogenicity test

To demonstrate the pathogenicity, purified FOC and SR were multiplied on sorghum grains. The field soil was sterilized using an autoclave for 30 min on 3 consecutive days at 121.6°C. A total of 25 g (25g) of inoculum from each pathogen were added to separate earthen pots. Five healthy chickpea seeds of the susceptible variety PBG 7 (Punjab Agricultural University, Punjab, India) were treated with a 1% sodium hypochlorite solution for surface disinfection, and each pot contained three replications.

The pots without inoculums were assigned to the control group. Symptoms of the disease were observed for up to 25 days. The pathogen was re-isolated and compared with the original culture after harvesting plants exhibiting signs of collar rot (Meena et al., 2024).

#### 2.7.1 Soil preparation and experimental setup

Soil collected from the LPU agricultural field (31.2560° N, 75.7051° E) was sterilized using 70% formalin and placed in earthen pots (5 kg/pot). The effectiveness of sterilization was verified through microbial load assessment before and after treatment using the plate-counting method (Debode et al., 2024). After 1 week of sterilization, pathogen inoculums were introduced into the pots (5 g/kg soil) and kept for 15 d to create sick soils. After this process, *Trichoderma* (2 × 10^7^ cfu/mL) and BC (1%) were applied to the soil, either individually or in combination, as amendments. The experimental design included the following treatment plan: (a) FOC alone, (b) SR alone, (c) *T. harzianum* alone, (d) BC 1% alone, (e) FOC+ BC (after sowing), (f) SR + BC (after sowing), (g) FOC + *T. harzianum* (soil application), (h) SR + *T. harzianum* (soil application), (i) FOC +BC + *T. harzianum* (soil application), and (j) SR + BC + *T. harzianum* (soil application) for amended soil in chickpea cultivation. Each treatment was replicated three times in a completely randomized design (CRD) (Ahmad et al., 2024).

#### 2.7.2 Application of *T. harzianum*

*T. harzianum* was cultivated on PDA in Petri dishes at 28°C for 5 days to obtain conidial suspensions. The conidial suspension was prepared by collecting spores from the culture surface using sterilized water and a spatula; these conidia were then immersed in Tween 20 (0.05%). The *Trichoderma* spore count was conducted using a hemocytometer and maintained at 1 × 10^7^/mL (Khaledi and Taheri, 2016). The chickpea variety PBG 7 seeds were procured from Punjab Agricultural University, Ludhiana, Punjab, India, and surface sterilized using sodium hypochlorite (1% NaOCl) (Davoudpour et al., 2020). After sterilization, these chickpea seeds were soaked in a 10 mL conidial suspension (1 × 10^7^/mL) of *Trichoderma* along with carboxymethyl cellulose (2% CMC) and kept on a rotary shaker (Thermo Fisher Scientific, USA) at 250 rpm for 30 min. The uniformly primed chickpea seeds were subsequently air-dried under sterile conditions in a laminar airflow cabinet (Thermo Fisher Scientific, USA) (Larena et al., 2010). For the application of *Trichoderma* to soil, conidial suspensions were mixed with BC-amended soils in the pots.

### 2.8 Statistical analysis

All the research data were analyzed using R Software (v 4.2.3) with one-way ANOVA to assess treatment differences (*p* < 0.05). LSD and Duncan's tests were applied for multiple comparisons (*p* < 0.05, *p* < 0.1). Data were organized in Microsoft Excel 2019, and graphs were generated using Origin Pro (v 10.10.178).

## 3 Results

### 3.1 Molecular characterization of *Trichoderma* isolates

**Figure 1:**
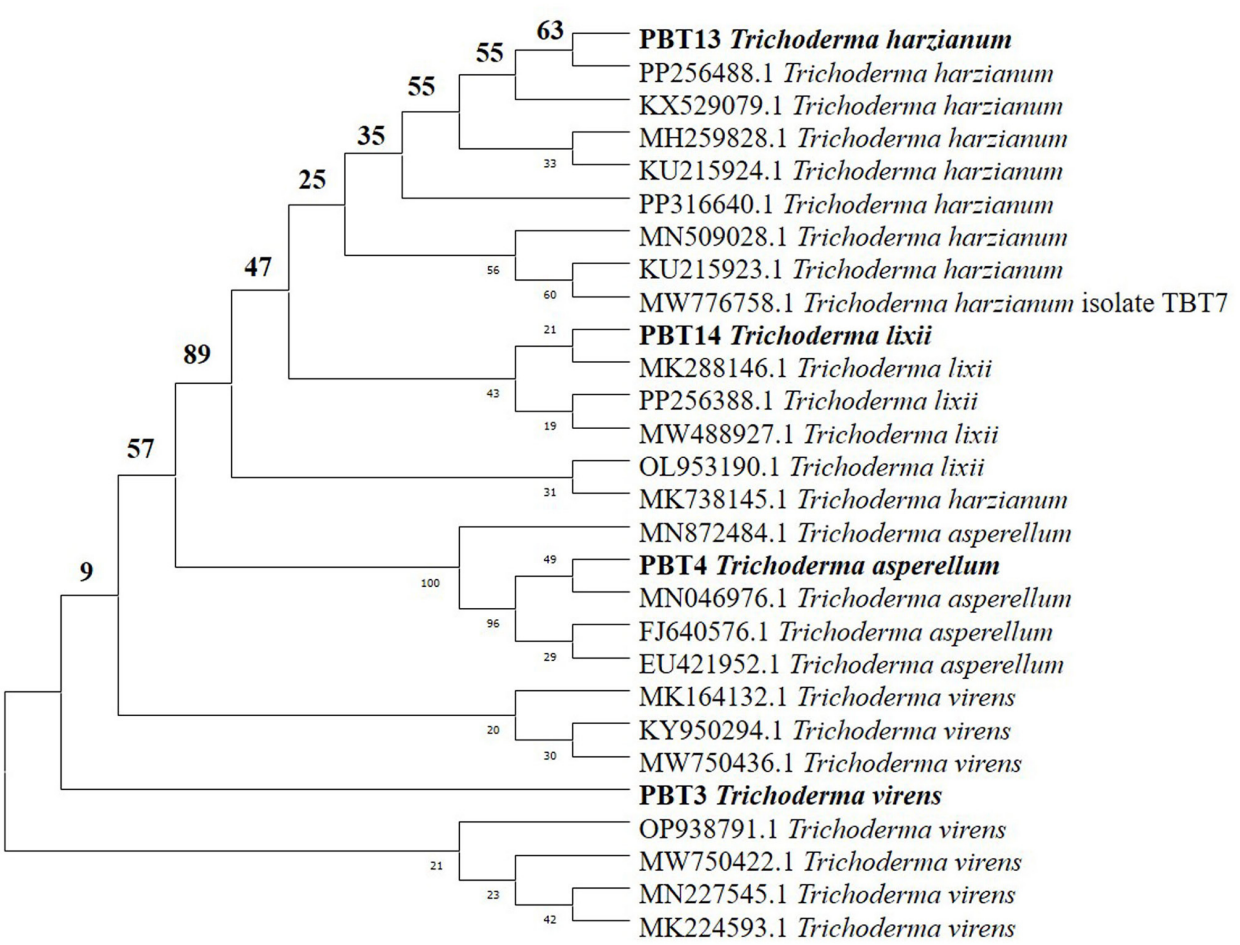
Phylogenetic tree of four *Trichoderma* spp.

Out of the 21 *Trichoderma* accessions collected for the selection of the BCA with biocontrol activity against two notorious soil-borne pathogens in chickpea plants, four strains were distinctly identified at the species level. PCR amplification and sequencing of the ITS region utilizing ITS1 and ITS4 primers enabled the molecular identification of different *Trichoderma* isolates (Table 1). For partial genome sequencing, the amplified products, ~500 and 600 bp in length, were submitted to Mr. Biologist (Pune, India). The sequences obtained were then sent to the NCBI database for verification. Complete similarity (100%) of the *Trichoderma* isolates *T. harzianum* (accession code: PP256488), *T. asperellum* (accession code: PP256386), *T. virens* (accession code: 0N678281), and *T. lixii* (accession code: PP256388) with strains cataloged in NCBI was observed. MEGA 7.1 software was used to generate a phylogenetic tree (Figure 1) using the neighbor-joining algorithm based on ITS sequence analysis, illustrating the genetic relationships among these *Trichoderma* isolates. The phylogenetic assessment of the four strains of *Trichoderma*, as depicted in the dendrogram, revealed a high level of closeness among *T. harzianum, T. virens, T. lixii*, and *T. asperellum*. These strains exhibited a close genetic relationship with other members of the same genus (Kumari et al., 2024).

**Table 1:** Molecular characterization of *Trichoderma* spp.

**S.no**.	**Primers**	**Amplicon size (bp)**	**Species**
1	ITS1 (forward primer): 5′-TCCGTAGGTGAACCTGCGG-3′ITS4 (reverse primer): 5′-TCCTCCGCTTATTGATATGC-3′	580	*T. lixii*
2	ITS1 & ITS4	650	*T. asperellum*
3	ITS1 & ITS4	590	*T. harzianum*
4	ITS1 & ITS4	600	*T. virens*

### 3.2 SEM analysis of the biocontrol property of *Trichoderma*

**Figure 2:**
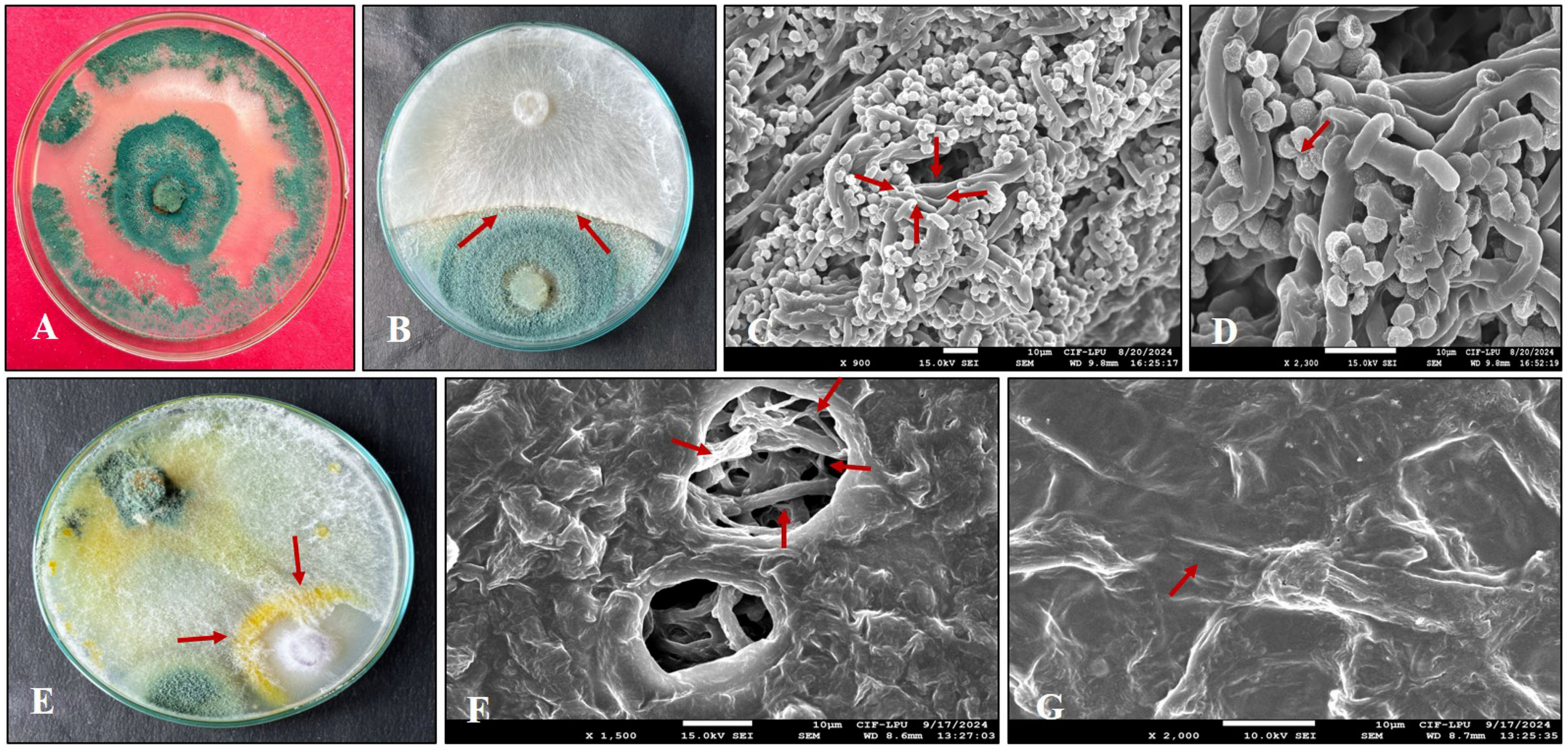
Scanning electron microscopy (SEM) analysis of the hyphal interaction between *Trichoderma* and phytopathogens. **(A)** Pure culture of *Trichoderma harzianum*; **(B)** Zone of inhibition (arrowhead) observed between *T. harzianum* and SR; **(C)** *Trichoderma* penetrating the hyphae of SR; **(D)** Hyphae of *Trichoderma* coiling around SR; **(E)** Zone of inhibition (arrowhead) noted between *T. harzianum* and FOC; **(F)** Hyphae of FOC damaged (arrowhead) by *T. harzianum*; **(G)** *T. harzianum* coiling (arrowhead) around the hyphae of FOC.

Microscopic investigation of fungal pathogens (*Fusarium* and *Sclerotium*) in dual culture revealed that *Trichoderma* (Figure 2A) made hyphal contact with the phytopathogens within 3 to 4 days of inoculation. *T. harzianum* was identified as the potent antagonist, as it grew over both phytopathogens. SEM analysis and dual culture exhibited a similar pattern of antagonist-pathogen interactions. SEM observations of the interactions between *Trichoderma* and the phytopathogens (Figures 2B, C) demonstrated two modes of antagonism by *Trichoderma*. Following contact, *T. harzianum* hyphae tightly coiled around the hyphae of *Fusarium* and *Sclerotium*, resulting in a wrinkled appearance or causing them to collapse (Figures 2D, G). In another mode, the hyphal tips of *Trichoderma* penetrated the pathogen hyphae (Figure 2E), creating holes that led to their collapse (Figures 2C, F). Additionally, the pathogen colonies completely disintegrated after 7 days of inoculation, and a large number of *Trichoderma* conidia were produced.

### 3.3 FTIR analyses

**Figure 3:**
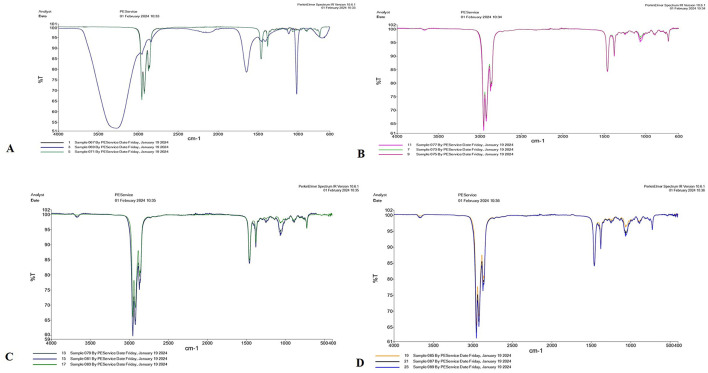
FTIR spectra of four *Trichoderma* species: **(A)** *T. virens*, **(B)** *T. lixii*, **(C)** *T. harzianum*, and **(D)** *T. asperellum* analyzed between 650 and 4,000 cm^−1^.

The aforementioned four species of *Trichoderma* were characterized by FTIR. In *T. virens*, the peak observed at 3,271.96 cm^−1^ indicates hydroxyl (OH) functional groups, typically found in carboxylic acids, phenols, or alcohols. The peaks at 2,956.23 cm^−1^ and 2,924.78 cm^−1^ correspond to C-H stretching vibrations, which are typically present in alkanes or alkyl groups. The peak at 1,643.95 cm^−1^ suggests amide I and II bands, indicating protein content. A peak at 1,114.04 cm^−1^ is characteristic of C-O-C stretching vibrations, indicating carbohydrate components (Figure 3A). In *T. lixii*, peaks within the 2,850–2,950 cm^−1^ range indicate C-H stretching vibrations, typically associated with alkane groups. The peak at 1,650 cm^−1^ suggests C=O stretching, which is indicative of carbonyl groups commonly found in proteins and lipids. This species also exhibited unique peaks between 1,100–1,250 cm^−1^, likely due to the presence of chitin, hemicelluloses, and β-glucans in the cell wall (Figure 3B). In *T. harzianum*, peaks observed ~2,850–2,950 cm^−1^ correspond to C-H stretching vibrations, typically associated with aliphatic chains in lipids. The peaks in the 1,200–1,000 cm^−1^ region are linked to C-O and C-O-C bond elongation modes, which are characteristic of cellulose and chitin, key components of fungal cell membranes (Figure 3C). In *T. asperellum*, the peaks between 2,800 and 2,900 cm^−1^ are characteristic of C-H stretching, while the peak at 1,066.01 cm^−1^ is related to C-O-C stretching vibrations, indicative of carbohydrates and esters. FTIR spectra also show peaks ~2,920 cm^−1^ (C-H stretching), which may vary in intensity due to its enhanced ability to degrade hydrocarbons and other organic pollutants, as well as the presence of stress response proteins (Figure 3D). Unique protein peaks ~1,650 and 1,550 cm^−1^ in *T. asperellum* could become more pronounced due to the production of stress response proteins under specific environmental conditions (Cantika et al., 2023). In *T. virens*, secondary metabolites appear as peaks near 1,730 cm^−1^, indicative of C=O stretching in ester compounds. FTIR spectra may exhibit distinct peaks related to these secondary metabolites, which play roles in plant growth enhancement and act as biological control agents (Racić et al., 2023). Variations in amide II and I bands ~1650 and 1550 cm^−1^ may reflect differences in the enzymatic protein profiles involved in biocontrol and organic material degradation (Miyano et al., 2020).

The integration of ITS sequencing, FTIR spectroscopy, and VOC profiling provides a robust approach for *Trichoderma* identification: ITS sequencing ensures differentiation of closely related fungal species, FTIR spectroscopy provides biochemical insights (functional groups), and VOC profiling captures metabolic signatures. Together, these methods combine genetic, biochemical, and metabolic data for inclusive strain differentiation. This multifaceted approach boosts the accuracy and reliability of *Trichoderma* identification (Druzhinina et al., 2005; Santos et al., 2010; Zeppa et al., 2020).

### 3.4 Characteristics of BC

BC used in the research experiments was produced from hardwood biomass through pyrolysis at 500 °C. The essential properties of the resulting BC were as follows: pH, 7.68; surface area, 73.5 m^2^/g; total nitrogen (N), 2.73%; carbon (C), 76.5%; potassium (K), 4.30%; and phosphorus (P), 0.21%.

### 3.5 Direct effect of BC and *Trichoderma* on phytopathogens in lab conditions

**Figure 4:**
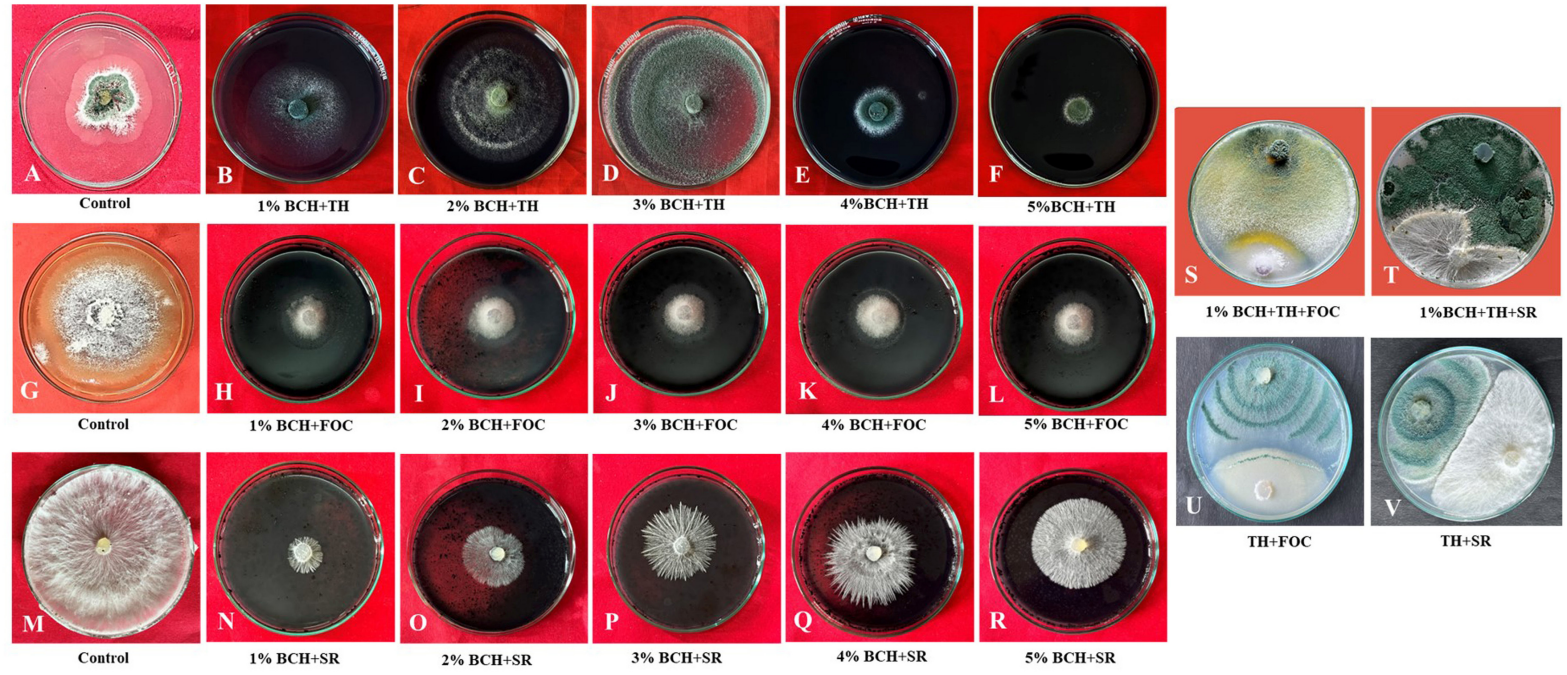
Evaluation of mycelial growth in *T. harzianum* and phytopathogens (FOC and SR) using various concentrations of biochar (1%−5%). **(A, G, M)** Control of *Trichoderma*, FOC, and SR; **(B–F)** Effect of different concentrations of biochar (1%−5%) on the growth of *T. harzianum;* **(H–L)** Effect of different concentrations of biochar (1%−5%) on the growth of FOC; **(N–R)** Effect of different concentrations of biochar (1%−5%) on the growth of SR; **(S, T)** Dual culture of *Trichoderma* and phytopathogens (FOC and SR) on 1% biochar-fortified media (potato dextrose agar); **(U, V)** Dual culture of *Trichoderma* and phytopathogens (FOC and SR) on potato dextrose agar.

The addition of biochar (BC) to the PDA culture medium significantly influenced the mycelial growth of phytopathogens (Figure 4). The dual culture technique involving *Trichoderma harzianum*, BC, and the soil-borne phytopathogens FOC and SR showed notable suppression of pathogen proliferation. Different concentrations of BC (1–5%) reduced the growth radius of both soil-borne pathogens (FOC and SR) in separate experiments. In both experiments, the mycelial growth rate in the nutrient medium declined due to suppression caused by varying concentrations of BC compared to the control. In the biochar-fortified media, mycelium growth decreased, while in the non-amended medium (control), the fungal pathogen fully colonized the *Petri* dish. Thus, the hardwood biochar-enriched media inhibited the growth of both phytopathogens, indicating a direct impact on the fungal pathogens (Figure 4).

The study evaluates the effect of different concentrations of BC (1 to 5%) on the radial growth of three organisms: *Fusarium oxysporum* (FOC), *Sclerotium rolfsii* (SR), and *Trichoderma harzianum*. The results indicate significant differences in the growth inhibition of the pathogens FOC and SR, as well as in the growth of the beneficial fungus *T. harzianum*. The highest inhibition of FOC was observed at the 1% BC treatment, with an inhibition percentage of 86.67 ± 1.4% (Table 2). BC suppresses pathogen growth by changing soil properties such as pH, providing nutrient availability for beneficial microbes, influencing root exudates, and activating ISR (induced systemic resistance). The concentration can inhibit organism growth due to several factors. As the concentration of BC increased, the inhibition gradually decreased. At 5% BC, the inhibition was reduced to 64.17 ± 1.4%. This indicates that higher concentrations of BC are less effective in inhibiting the growth of FOC. Similarly, SR exhibited the highest inhibition at the 1% BC concentration, with an inhibition percentage of 88.14 ± 1.3%. As the BC concentration increased, the inhibition dropped significantly to 31.31 ± 2.2% at the 5% concentration, demonstrating a strong inverse relationship between BC concentration and SR inhibition. The radial growth of *T. harzianum* was highest at the 1% BC concentration, measuring 4.43 ± 13.3 cm. As BC concentration increased, the growth of *T. harzianum* gradually decreased, reaching 0.50 ± 1.5 cm at the 5% BC concentration. However, even at higher concentrations, *T. harzianum* still showed some growth, indicating that BC is less inhibitory to beneficial fungi. In the control group (without BC), there was no inhibition of FOC or SR, and the radial growth of *T. harzianum* was 2.10 ± 6.3 cm, suggesting that BC has a measurable impact on both pathogens and beneficial fungi. The results were statistically analyzed, revealing notable differences among the treatments across all three organisms. The critical difference (CD) values for FOC, SR, and *T. harzianum* were 2.43, 5.97, and 0.148, respectively. The standard error (SE) of the mean values for FOC, SR, and *T. harzianum* were 0.75, 1.83, and 0.06, respectively, indicating precision in the measurements. These findings show that the concentration of BC has a specific effect on the growth of both beneficial and phytopathogenic fungi.

**Table 2:** Effect of biochar on the radius growth (cm) of *Fusarium oxysporum* (FOC), *Sclerotium rolfsii* (SR), and *T. harzianum*.

**Sr. no**.	**Treatments (%)**	***Fusarium oxysporum***	**% inhibition**	***Sclerotium rolfsii***	**% inhibition**	***T. harzianum***
1.	1	0.53 ± 0.058^f^	86.67 ± 1.4^a^	0.5 ± 0.05^f^	88.14 ± 1.3^a^	4.43 ± 13.3^a^
2.	2	0.83 ± 0.05^e^	79.17 ± 1.4^b^	0.95 ± 0.05^e^	79.26 ± 1.3^b^	3.35 ± 10.05^b^
3.	3	1.00 ± 0.0^d^	75.00 ± 0^c^	1.46 ± 0.05^d^	67.40 ± 1.3^c^	2.95 ± 8.85^c^
4.	4	1.23 ± 0.06 ^c^	69.17 ± 1.4^d^	2.3 ± 0.32 ^c^	47.40 ± 7.1^d^	1.25 ± 3.75^e^
5.	5	1.43 ± 0.05^b^	64.17 ± 1.4^e^	3.1 ± 0.1^b^	31.11 ± 2.2^e^	0.50 ± 1.5^f^
6.	control	4.00 ± 0.0^a^	0	4.5 ± 0.28^a^	0	2.10 ± 6.3^d^
	CD	0.07	2.43	0.19	5.97	0.148
	SE (m)	0.02	0.75	0.06	1.83	0.06
	SE (d)	0.03	1.73	0.09	2.59	0.06

### 3.5 Pathogenicity of phytopathogens

The pathogenicity of FOC and SR was assessed on chickpea (PBG7) using an artificial soil inoculation method. In Fusarium wilt, the infection begins on the leaves, resulting in partial yellowing, wilting, and ultimately plant death. Similarly, in collar rot, infection starts at the collar region, leading to chlorosis, and stem rot. The results showed that FOC exhibited the highest disease incidence (73%), followed by SR (60%).

### 3.6 Synergistic effects of *Trichoderma* and BC on chickpea growth and disease control

**Figure 5:**
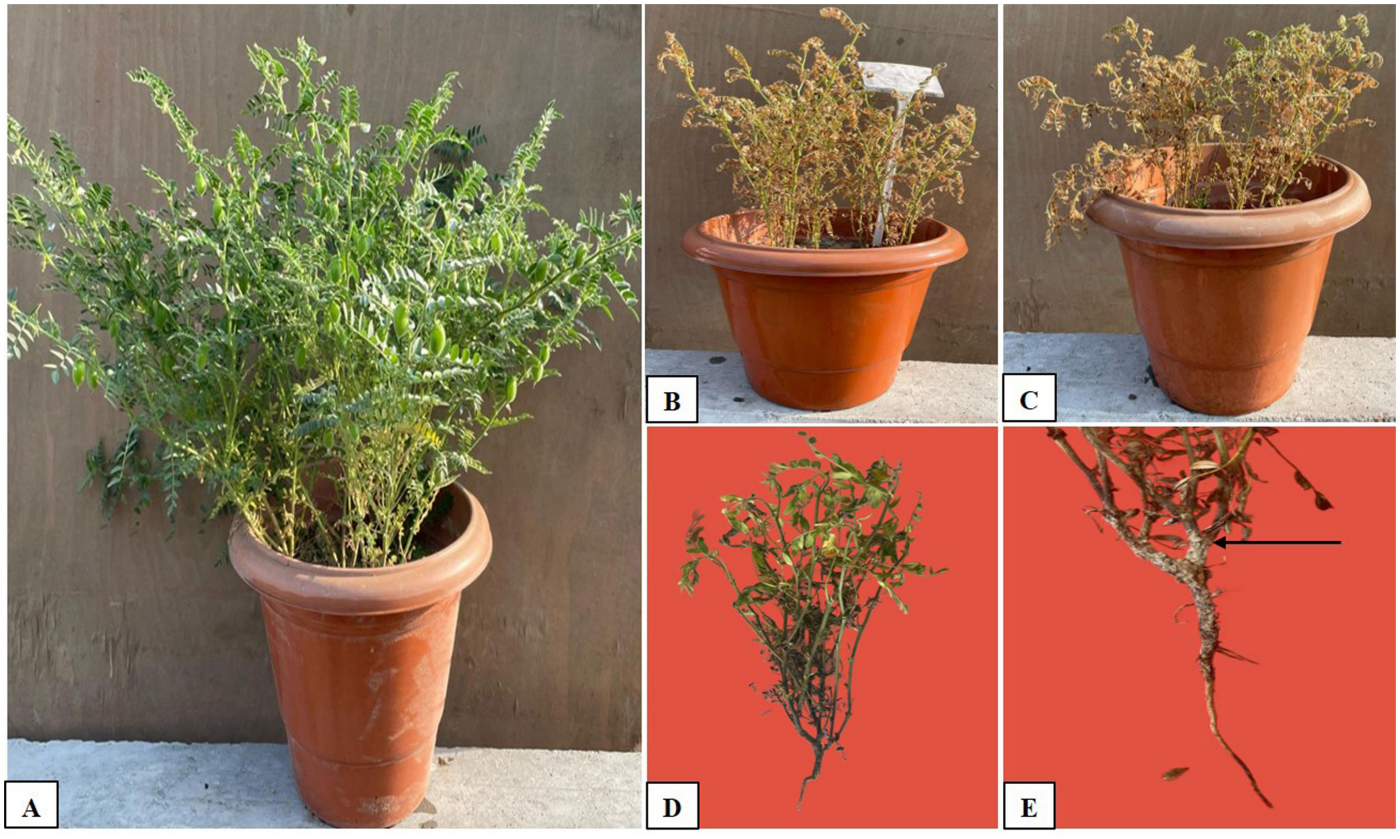
Growth of chickpea seeds in pots with and without treatment: **(A)** Full growth in the pot with soil mixed with biochar + *Trichoderma*, **(B)** Control (FOC), **(C)** Control (SR), **(D)** Fusarium wilt caused by *Fusarium oxysporum*, and **(E)** Collar rot caused by *Sclerotium rolfsii*, as indicated by the arrow.

**Figure 6:**
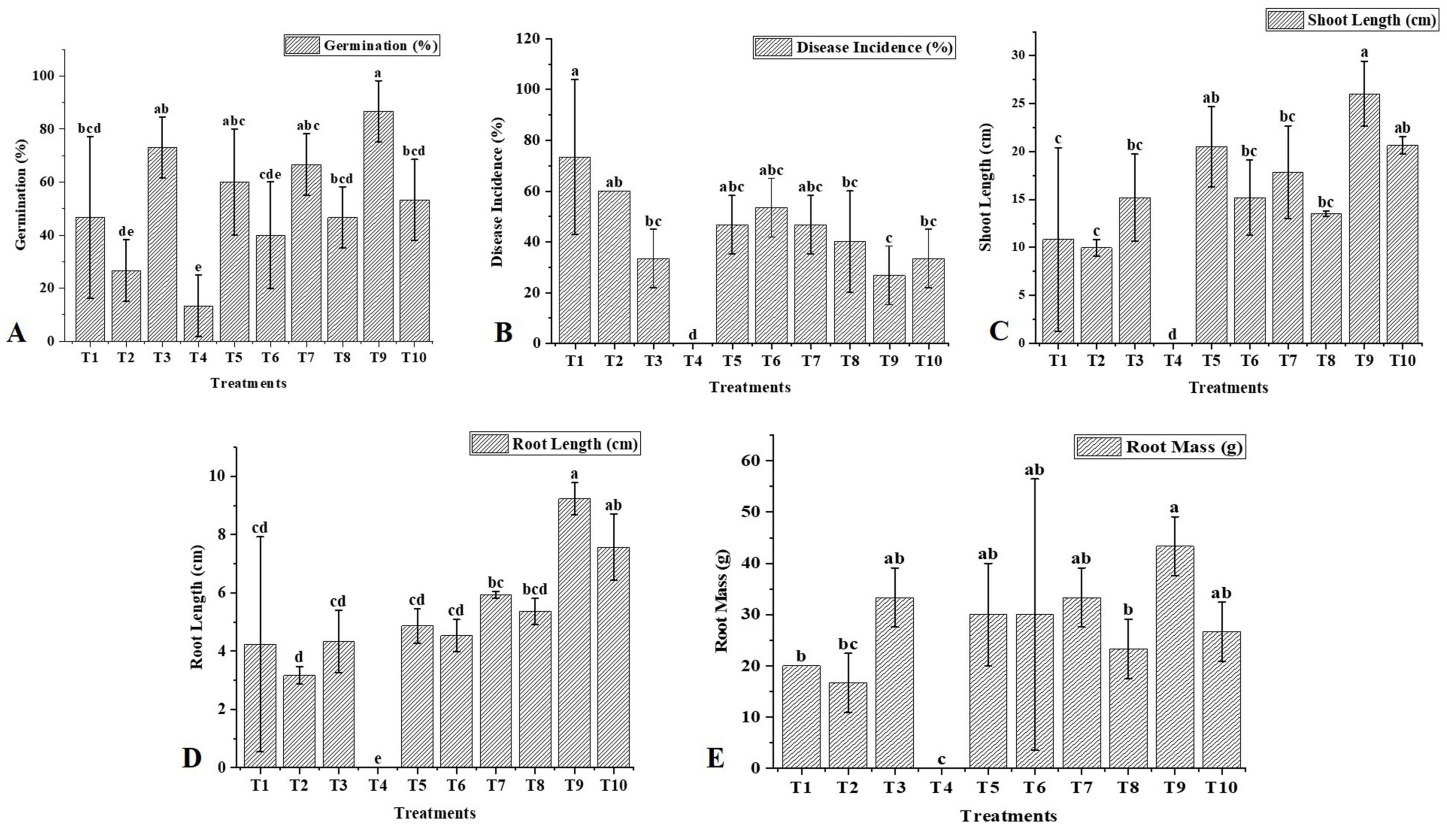
Effect of *T. harzianum* and biochar on the growth attributes and disease incidence of chickpea. **(A)** Treatment effects on germination (%), **(B)** Treatment effects on disease incidence (%), **(C)** Treatment effects on shoot length (cm), **(D)** Treatment effects on root length (cm), and **(E)** Treatment effects on root mass (g). The letters above the bars indicate statistical differences between treatments. Values with different letters at the top show significant differences (*P* ≤ 0.05), as determined by the LSD test.

In this study, we investigated the indirect and direct effects of *Trichoderma* and BC on phytopathogens in chickpea plants. For the direct effect, chickpea seeds were sown in earthen pots (clay pots) and inoculated separately with two soil-borne pathogens (FOC and SR) (Figure 5). A decline in germination percentage, shoot and root length, and root mass of the plants, along with increased disease incidence, was observed, and disease incidence was calculated using the equation shown below. At the initial stage of growth, some plants exhibited unusual symptoms, such as yellowing, drooping, stunting, and wilting. The germination percentage using T_1_ (FOC) in chickpeas was observed to be 47% ± 30.55%, whereas in T_2_ (SR), T_3_ (TH), and T_4_ (BC), it was 27 ± 11.55%, 73 ± 11.55%, and 13 ± 11.55%, respectively (Table 3). *Trichoderma* treatment (FOC+TH) against FOC reduced disease incidence in chickpeas to 40 ± 10.00% compared to the control (FOC), which was 73 ± 5.77%. Similarly, *Trichoderma* treatment (SR+TH) against SR reduced disease incidence to 43.33 ± 5.77% compared to the control (SR), which was 60 ± 20.00%. Treatment with biochar (FOC+BC) reduced *Fusarium* wilt disease incidence to 46.66 ± 5.77% compared to the control (FOC), which was 73 ± 5.77% (Table 3). Additionally, the same treatment reduced collar rot disease incidence to 53.33 ± 15.27% compared to the control (SR), which was 60% (Table 3). The indirect effects of *Trichoderma* aid in maintaining nutrient cycling and help plants become more resilient to biotic and abiotic stress, while BC increases the diversity and activity of microbes in the soil, enhancing soil fertility, improving the root system, and supporting plant health (Paveen et al., 2024). The combined effects of BC and *Trichoderma* improved chickpea crop growth and soil health. Each treatment with pathogens (FOC and SR) led to a considerable decrease in shoot length, root length, and root mass (Table 3; Figure 6). However, the maximum root length, shoot length, and root mass were recorded in treatment T9 (9.23 cm, 26.03 cm, and 43.33 g), where the combined application of *Trichoderma* with BC (@3%) was used. In contrast, the minimum values for these metrics were observed in T1 (FOC) and T2 (SR) treatments (4.23 cm, 3.17 cm and 10.83 cm, 9.97 cm and 20.00 g, 16.67 g) under disease stress conditions, respectively. The disease incidence was measured as follows:

**Table 3:** Efficacy of *Trichoderma* and biochar on chickpea growth, development, and disease incidence.

**Sr no**.	**Treatments**		**Germination%**	**Shoot length**	**Root length**	**Root mass**	**Disease incidence %**
1	T1 (control)	FOC	47.00 ± 30.55^bcd^	10.83 ± 9.58^c^	4.23 ± 3.69 ^cd^	20.00 ± 0.00^b^	73.00 ± 5.77^a^
2	T2 (control)	SR	27.00 ± 11.55^de^	9.97 ± 0.84^c^	3.17 ± 0.31^d^	16.67 ± 5.77^bc^	60.00 ± 20.00^ab^
3	T3 (control)	TH	73.00 ± 11.55^ab^	15.20 ± 4.54^bc^	4.33 ± 1.07^cd^	33.33 ± 5.77^ab^	00.00 ± 0.00^f^
4	T4 (control)	BC	13.00 ± 11.55^e^	0.00 ± 0.00^d^	0.00 ± 0.00^e^	0.00 ± 0.00^c^	0.00 ± 0.00^f^
5	T5	FOC + BC	60.00 ± 20.00^abc^	20.50 ± 4.16^ab^	4.87 ± 0.59^cd^	30.00 ± 10.00^ab^	46.66 ±5.77 ^bcd^
6	T6	SR+ BC	40.00 ± 20.00^cde^	15.20 ± 3.94^bc^	4.53 ± 0.55^cd^	30.00 ± 26.46^ab^	53.33 ±15.27^bc^
7	T7	FOC + TH	67.00 ± 11.55^abc^	17.83 ± 4.85^bc^	5.93 ± 0.12^bc^	33.33 ± 5.77^ab^	40.00 ± 10.00^cde^
8	T8	SR + TH	47.00 ± 11.55^bcd^	13.53 ± 0.25^bc^	5.37 ± 0.45^bcd^	23.33 ± 5.77^b^	43.33 ±5.77^bcde^
9	**T9**	**FOC+** **BC** **+** **TH**	**87.00** **±11.55**^**a**^	**26.03** **±3.41**^**a**^	**9.23** **±0.55**^**a**^	**43.33** **±5.77**^**a**^	**27.00 ± 5.77** ^**e**^
10	T10	SR+ BC + TH	53.00 ± 15.28^bcd^	20.63 ± 0.91^ab^	7.57 ± 1.14 ^ab^	26.67 ± 5.77^ab^	33.00 ± 11.54^de^
		CD Value	31.78	7.497	2.276	18.008	16.73
		SE(m)	10.60	2.504	0.760	6.014	5.63
		SE(D)	14.99	3.541	1.075	8.506	7.97

FOC, Fusarium oxysporum f.sp. ciceri, SR, Sclerotium rolfsii, TH, Trichoderma harzianum, BC, Biochar.

Disease incidence (%) = no of diseased plants × 100/total number of plants examined.

## 4 Discussion

In this study, we isolated 21 unidentified strains of *Trichoderma* spp. obtained from the rhizosphere of uncultivated fields in Punjab, India, based on evidence that these isolates may serve as potent BCAs and be beneficial for crops and soil health. Biochemical assays revealed that these isolates produce cell wall-degrading enzymes (e.g., chitinase and β-1,3-glucanase) and exhibit antagonism against two soil-borne phytopathogens, *Fusarium oxysporum* f.sp. *ciceri* and *Sclerotium rolfsii* (Kabir et al., 2023). Out of the 21 strains, four were confirmed as four different species of *Trichoderma:* PBT9 (*T. lixii*), PBT13 (*T. harzianum*), PBT4 (*T. asperellum*), and PBT3 (*T. virens*) through PCR amplification, sequencing, and sequence analysis of the ITS1 and ITS4 regions of *Trichoderma* (Kumari et al., 2024). The ITS region is one of the most reliable genetic loci for developing specific primers capable of distinguishing between closely related fungal spp. (Shahid et al., 2014). Several reports have indicated that the internal transcribed spacer (ITS) region serves as a universal barcode for the accurate characterization of *Trichoderma* isolates (Mazrou et al., 2020; Matas-Baca et al., 2022). According to the results, amplification of the rDNA region in four isolates revealed distinct polymorphic bands at ~636 and 657 bp in isolates T7 and T3, respectively. Molecular characterization of these bands revealed that they correspond to distinct isolates, representing *T*. *lixii* (580 bp), *T. harzianum* (590 bp), *T. asperellum* (650 bp), and *T. virens* (600 bp), respectively. Similar findings have been reported in previous studies (Ru and Di, 2012; Shobha et al., 2020; Matas-Baca et al., 2022).

The fungal cell wall comprises chitin, β-(1,3)-glucan, and β-(1,4)-linked N-acetylglucosamine, integrated within an irregular matrix that includes galactomannans, α-glucans, and other carbohydrate polymers (Gautam et al., 2024). These polymers contain various functional groups that facilitate metal binding. The presence and functionality of these polymers in metal binding were confirmed using FTIR spectroscopy in this study (Shoaib et al., 2013). *Trichoderma*, recognized for its biocontrol properties and promotion of plant growth, includes several morphologically similar species, making traditional identification methods challenging. This research compares FTIR-ATR spectroscopy with molecular techniques to assess its effectiveness in differentiating between *Trichoderma* species.

FTIR-ATR spectroscopy provided distinct spectral signatures for different *Trichoderma* species, indicating its potential for precise identification (Vieira et al., 2024). This method reduced sample preparation time and offered a rapid, cost-effective alternative to traditional molecular techniques. Specific absorbance peaks corresponding to functional groups (e.g., hydroxyl groups, C-H stretching, amide bands) were identified for each *Trichoderma* species. *T. virens, T. asperellum, T. lixii*, and *T. harzianum* displayed unique spectral features, enabling differentiation based on their biochemical compositions. Various solvents (50% alcohol, ultrapure water, and 99.5% alcohol) were tested for sample preparation, with observable effects on spectral resolution. In our study, FTIR-ATR spectroscopy was utilized to characterize four *Trichoderma* strains: *T. harzianum, T. asperellum, T. lixii*, and *T. virens*. The spectral data, spanning the mid-infrared range (600 to 4,000 cm^−1^), revealed distinct peaks associated with functional groups indicative of various biomolecules. Dorjee also discussed functional groups in his study (Dorjee et al., 2024). Similarly, *T*. *asperellum* and *T*. h*arzianum* exhibited peaks corresponding to alkanes, carbohydrates, and cell wall components such as chitin. Additionally, *T. harzianum* displays interactions with nanoparticles, including ZnO, AgNP-TS, and CuO. Jurić et al. (2019) also addressed this in their study (Narware et al., 2023). The use of FTIR-ATR in this context demonstrates its capability for species differentiation as well as detailed biochemical analysis. This technique can complement molecular methods, offering a comprehensive approach to fungal identification and contributing to advancements in agricultural biotechnology and plant pathology. The protein and polysaccharide profiles can influence the efficacy of *Trichoderma* species as BCAs against plant pathogens. Species with higher polysaccharide content, such as *T. asperellum*, might exhibit stronger opposing effects because they release extracellular enzymes (Kubicek et al., 2014). Variations in protein content and structure suggest differing capabilities for enzyme production. *T. harzianum* and *T. virens*, known for their prominent amide bands, may be better suited for applications requiring robust enzyme activity. The lipid content, indicated by the C-H stretching region, can affect the ability of *Trichoderma* species to degrade hydrophobic pollutants. *T. virens*, with its higher lipid content, may be more effective in such applications (Jurić et al., 2019).

The GC-MS analysis of *T. asperellum, T. virens, T. harzianum*, and *T. lixii* identified a diverse range of VOCs, emphasizing their role in biocontrol and promoting plant growth. These metabolites include hydrocarbons, alcohols, ketones, esters, terpenes, and phthalates, each contributing to *Trichoderma*'s antifungal, antibacterial, and plant-growth-promoting properties (Kumar et al., 2025). Hydrocarbons and alcohols, such as hexadecane, nonane, and heptadecane, were found in multiple strains and are known to enhance fungal defense and environmental adaptability. The presence of 2,3-butanediol and 1-heptadecanol in *T. virens* and *T. harzianum* suggests their role in promoting plant growth and inducing systemic resistance. Additionally, nonanal in *T. harzianum* has been linked to the activation of plant defense mechanisms. Ketones and esters, including dehydroacetic acid and sorbic acid vinyl ester, indicate possible antifungal properties, as they can inhibit the germination of fungal spores (Kumari et al., 2024). The detection of phthalic acid derivatives across multiple strains suggests antimicrobial activity, contributing to pathogen suppression. Terpenes and aromatics such as turmerone, curlone, and 7-tetradecene are predominantly found in *T. harzianum* and *T. virens*, reinforcing their strong antifungal and antibacterial potential. Additionally, phenol derivatives like 3,5-dimethyl-phenol enhance these antimicrobial capabilities. The presence of phthalates and siloxanes, including dibutyl phthalate and diamyl phthalate, suggests potential nematicidal and antifungal effects, although further investigation is needed to confirm their biological significance (Li et al., 2018).

In our research, we also examined the hyphal interaction between *T. harzianum* and FOC and SR. SEM analysis showed that *Trichoderma* coiled around the hyphae of both phytopathogens at certain locations and penetrated them. It is well documented that coiling is a common response of *Trichoderma* toward specific phytopathogens, and our results are consistent with those of Kishan et al. (2017). In this context, the antagonistic activity of *T. harzianum* against both pathogens was confirmed by the formation of additional structures, such as *Trichoderma* hyphae penetrating the hyphae of the pathogens, which leads to cell disruption and partially explains their role in controlling pathogens. A comparable observation was reported by Nofal et al. (2021).

The current study also demonstrated the positive effects of BC and *Trichoderma* on the growth parameters of chickpeas under field conditions. The co-application of *Trichoderma* and BC showed improved performance compared to single applications and the control. This improvement could be due to the synergistic effect of *T. harzianum* and BC, which significantly promoted vegetative growth compared to the untreated group. Previous findings indicated that *Trichoderma* spp. can enhance plant growth by colonizing roots, promoting foliar tissue growth, stimulating lateral root formation, and improving overall root structure (Sani and Yong, 2022). It has also been shown that *Trichoderma* strains colonize the plant root zone, increasing soil mycobiome diversity and significantly reducing the population of *Fusarium* spp. while promoting plant growth and nutritional status by secreting bioactive compounds in the rhizosphere (Abuhena et al., 2023; Tyśkiewicz et al., 2022).

BC is a reliable soil supplement that improves soil fertility, structure, and interactions with soil microbes (Alkharabsheh et al., 2021). It acts as a medium for growth development, nutrient uptake, and root colonization of beneficial fungi through hyphal growth and elongation because of its larger surface area (Hussain et al., 2021). Some studies have proven that BC enhanced the multiplication of *Trichoderma* in the soil amended with BC compared to non-amended soil (Graber et al., 2010; Ahmad et al., 2024). Hu et al. (2014) also documented that BC boosted the *Trichoderma* proportion by 14.5% in soil treated with BC compared to non-treated soil. Similarly, the current results showed that the BC combined with *Trichoderma* enhances the ability to overcome the negative impact caused by FOC and SR in chickpeas. Syuhada et al. (2016) found that BC alone does not provide sufficient nutrients for plant growth, necessitating the use of additional fertilizers. Therefore, in this study, the co-application of *Trichoderma* and BC may have worked synergistically, leading to increased root and shoot biomass in chickpeas and controlling the pathogens.

## 5 Conclusion

Biochar-based soil amendments can enhance plant defense and reduce emerging plant pathogenic pressure. The response of chickpea plants to the aggressive *Fusarium oxysporum* f.sp. ciceris (FOC) and *Sclerotinia sclerotiorum* (SR) were affected by the availability of biochar (BC) in the soil. The comparison indicated that the combined application of BC (1–3%) with *T. harzianum* was the most effective in controlling both studied phytopathogens. Furthermore, using *T. harzianum* with BC increased the *Trichoderma* population in the soil, which further stimulated plant growth and inhibited disease progression. Thus, the synergistic effect of *Trichoderma* spp. and BC represents a reliable, robust, eco-friendly, and cost-effective strategy for sustainable crop production. However, future research should prioritize optimizing different types of BC dosage along with other *Trichoderma* strains across various pathogen systems. This study also illustrates the potential of FTIR-ATR as a rapid, sensitive, and cost-effective method for characterizing and identifying various species of *Trichoderma*.

## Data Availability

The original contributions presented in the study are included in the article/supplementary material, further inquiries can be directed to the corresponding authors.
